# Treating amblyopia using altered reality enhances the fine‐scale functional correlations in early visual areas

**DOI:** 10.1002/hbm.26526

**Published:** 2023-11-06

**Authors:** Xue Dong, Lijuan Liu, Xinxin Du, Yue Wang, Peng Zhang, Zhihao Li, Min Bao

**Affiliations:** ^1^ CAS Key Laboratory of Behavioral Science, Institute of Psychology Chinese Academy of Sciences Beijing China; ^2^ Department of Psychology University of Chinese Academy of Sciences Beijing China; ^3^ Beijing Institute of Ophthalmology, Beijing Tongren Hospital Capital Medical University Beijing China; ^4^ State Key Laboratory of Brain and Cognitive Science, Institute of Biophysics Chinese Academy of Sciences Beijing China; ^5^ School of Psychology Shenzhen University Shenzhen Guangdong China; ^6^ Department of Psychiatry and Behavioral Sciences Emory University Atlanta Georgia USA

**Keywords:** adaptation, altered reality, amblyopia, functional connectivity, plasticity

## Abstract

Amblyopia is a developmental visual disorder that causes substantial visual deficits. Studies using resting‐state functional magnetic resonance imaging have disclosed abnormal brain functional connectivity (FC) both across long‐range cortical sites and within the visual cortex in amblyopes, which is considered to be related to impaired visual functions. However, little work has examined whether restoring the vision of amblyopes accompanies with an improvement of FC. Here in adult amblyopes and healthy participants, we compared their brain FC before and after an altered‐reality adaptation training. Before the training, the voxel‐wise FCs of amblyopia patients were substantially weaker than those of healthy control participants both within and across the early visual areas. After the training, visual acuities improved in amblyopes but not in the control participants. The effect kept strengthening in the subsequent month without further adaptation. Importantly, we observed enhanced voxel‐wise FC both within and across the early visual areas of amblyopes. Moreover, the enhancement continued for at least 1 month. These results suggest that the effective treatment can improve both the amblyopes' vision and functional connections in the visual cortex.

## Graphical abstract

Treating amblyopia using altered reality with the complementary patchwork adaptation effectively improved the visual acuities of amblyopes. Such paradigm also enhanced voxel‐wise functional connectivity both within and across the early visual areas of amblyopes, promoting the transmission and integration of visual information.

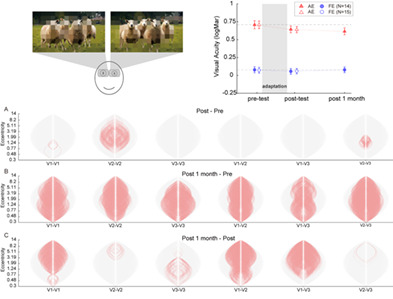

## 1 INTRODUCTION

Amblyopia is a common visual disorder that affecting 1%–6% of the population (McConaghy & McGuirk, 2019). Generally, it is characterized by visual deficiency of one eye, or infrequently both eyes, even after refractive correction (Holmes & Clarke, 2006). The impaired visual functions are considered to result from anisometropia, strabismus or other amblyogenic factors that interfere with normal development of the visual pathways during the first years of life (Birch, 2013; Birch & Kelly, 2023; Hunter & Cotter, 2018). Apart from low visual acuity, amblyopia is also accompanied by oculo‐motor deficits (Chung et al., 2015; Ghasia & Wang, 2022), extremely interocular imbalance (Ding, Klein, et al., 2013; Huang et al., 2011) and interocular suppression (Li et al., 2011; Mansouri et al., 2008), decreased contrast sensitivity (Bradley & Freeman, 1981), impaired motion perception (El‐Shamayleh et al., 2010), loss of stereoacuity (Levi et al., 2015), and other monocular and binocular visual dysfunctions (Hamm et al., 2014; Levi, 2020; Spang & Fahle, 2009).

Neuroimaging studies have demonstrated that visual deficits of amblyopia mainly arise from dysfunctions in the visual cortex (for review, see Joly & Frankó, 2014). It has been found that cortical activation in amblyopes showed a larger interocular difference than normal controls (Algaze et al., 2002). Furthermore, activation driven by stimuli presented in the amblyopic eye is reduced not only in the striate area (V1), but also in the extrastriate visual areas (V2–V4, MT/V5) (Bonhomme et al., 2006; Conner et al., 2007; Goodyear et al., 2000; Lerner et al., 2006), and even high‐order areas like face‐related fusiform (Lerner et al., 2003). Notably, it was reported that the cortical activation to stimuli in the amblyopic eye increased after perceptual training, which also led to the improvement of visual acuity (Zhai et al., 2013), suggesting that treatments for amblyopes could strengthen the stimulus‐driven neural responses.

Besides the abnormal stimulus‐elicited activation, spontaneous brain activity and functional connectivity (FC) also show striking differences between amblyopes and normal‐sighted participants, as revealed by resting‐state functional magnetic resonance imaging (rs‐fMRI). For example, the connectivity between mirrored regions in two hemispheres was found abnormal in amblyopes, indicating the impaired interhemispheric functional coordination (Liang et al., 2017). Seed‐based analysis has disclosed decreased FC between the primary visual cortex (Brodmann area 17) and areas of both the dorsal stream (e.g., middle temporal gyrus, superior occipital gyrus) and the ventral stream (e.g., lingual gyrus), showing the deficits of information transmission in the two visual pathways of amblyopes (Wang et al., 2014). Moreover, both visuo‐parietal FC and visuo‐cerebellum FC were found decreased, which was considered to be related to the defects in depth perception and visuomotor processing in amblyopes (Ding, Liu, et al., 2013). Recently, Mendola et al. (2018) conducted a fine‐scale analysis on the connectivity within early visual cortex (V1–V3) after dividing each visual area into sub‐regions representing visual fields of different eccentricities. Their findings revealed that as compared to normal‐sighted participants, the FC between iso‐eccentricity regions of interest (ROIs) in different early visual areas was decreased and the cross‐eccentricity FC was increased in participants with amblyopia. The abnormal FC was also associated with the poor visual acuity of the amblyopic eye.

**FIGURE 1:**
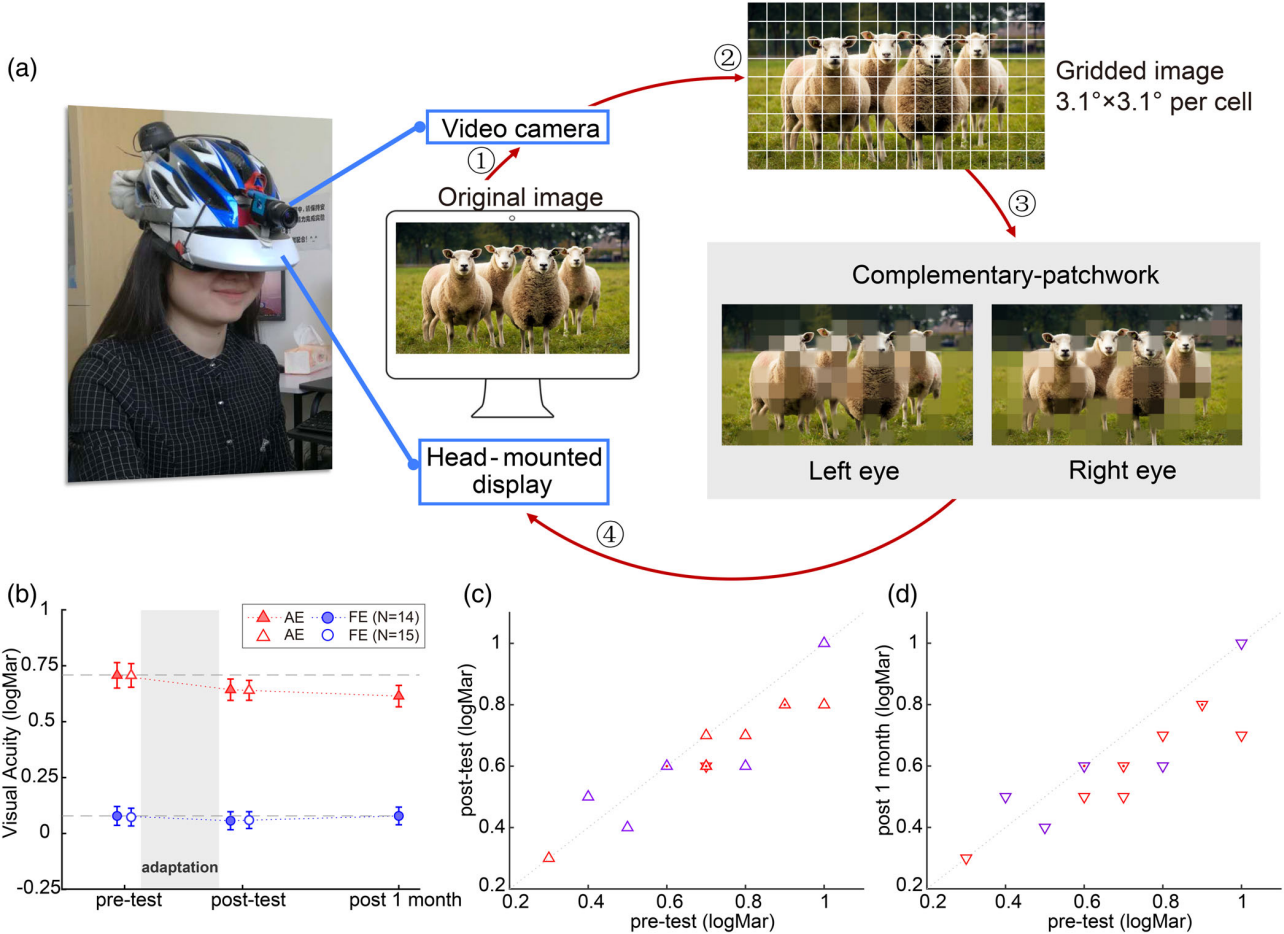
(a) Illustration of the devices for adaptation training. During adaptation, participants wore the altered‐reality system, which is comprised of a video camera and an HMD. At first, the camera captured images (original image) of what participants were looking at. The images were then divided into a 9 × 16 grid of square cells and made into two complementary images that were presented to two eyes respectively. (b) The visual acuity (measured with the ETDRS chart) of amblyopes who finished all three test sessions (filled triangles/circles) and who finished pre‐ and post‐tests (open triangles/circles). Error bars represent the standard error of mean. (c) Individual visual acuity of AE in the pre‐ and post‐tests. (d) Individual visual acuity of AE in the pre‐ and post‐1‐month tests. The result of each participant is denoted by a triangle (purple for patients with strabismus). The overlapped data points are presented with dots (two overlapped points) or hexagram (three overlapped points) for a clear presentation.

However, no work to date has examined the changes of brain FC following a treatment of amblyopia. Since the altered brain FC in amblyopia is also believed to be associated with impaired visual functions, effective training for amblyopia patients may contribute to the improvements of both their visual acuity and the brain FC. To address this issue, here we measured the FC of amblyopia patients and healthy control participants before and after they completed a training paradigm of complementary‐patchwork adaptation using altered reality (Figure 1a). Previously, our work revealed that complementary‐patchwork adaptation trainings could significantly improve visual acuity and contrast sensitivity of amblyopic eyes (Bao et al., 2018). In this study, we acquired the participants' rs‐fMRI data before, 1 day after, and 1 month after the training, respectively. We assessed how the training would affect the fine‐scale connections in early visual areas and also searched for any altered FC between visual areas and other brain networks. We hypothesized that effective training for the amblyopes could alter their brain FCs, especially for those within the visual cortex, promoting the transmission and integration of visual information.

## 2 METHOD

### 2.1 Subjects

Sixteen patients diagnosed with unilateral amblyopia (7 females, 9 males, mean age: 25.33 ± 6.62 years) and 14 healthy controls (7 females, 7 males, mean age: 25.79 ± 2.86 years) participated in the present study. Due to a scanner malfunction, the data of one patient were not included in the analysis and results. The clinical details of the other amblyopia patients were listed in Table 1. For patients with severe strabismus, the two screens of the goggles (see Hardware) may need to be specifically redesigned to provide extensive adjustability for the distance between the screens, which enables the misaligned eye to view the patchwork image that is matched to the image presented to the other eye. Therefore, when recruiting participants in this study, we did not consider any patient with strabismus angle larger than 5°. The control participants had no visual problems. All had normal or corrected to normal vision and matched in gender and age with the patients. All participants were right‐handed. The study protocol was approved by the Institutional Review Board of the Institute of Psychology, Chinese Academy of Sciences. Informed consent was obtained from all participants, in accordance with the Code of Ethics of the World Medical Association (Declaration of Helsinki).

**TABLE 1:** Clinical details of the patients.

Patient	Age (years)	Gender	Type of amblyopia	Pre‐test				Post‐test		Post 1 month	
				LE		RE		LE	RE	LE	RE
				Acuity (logMAR)	Refraction	Acuity (logMAR)	Refraction	Acuity (logMAR)	Acuity (logMAR)	Acuity (logMAR)	Acuity (logMAR)
01	15	Male	Anisometropia and strabismus (LE)	1	+6.00/+1.00 × 105	−0.1	+0.00	1	−0.2	1	−0.1
02	28	Female	Anisometropia (LE)	0.8	+4.75/+0.50 × 110	0	−1.00/−0.50 × 15	0.7	0.1	0.7	0.1
03	22	Male	Anisometropia and strabismus (LE)	0.8	+2.75	0	+0.00	0.6	0	0.6	0
04	18	Female	Anisometropia (LE)	0.7	+1.00/+1.75 × 105	0.1	+0.00	0.6	0	0.6	0.1
05	23	Female	Anisometropia (RE)	0	+1.00	0.7	+3.50/+0.50 × 70	0	0.7	0	0.6
06	20	Male	Anisometropia (LE)	0.7	+1.00/+0.75 × 95	0	−3.00/+0.75 × 145	0.6	0.1	Canceled due to COVID‐19	
07	21	Male	Anisometropia (RE)	0.5	−2.00/+0.50 × 105	1	−0.25/+4.50 × 75	0.4	0.8	0.4	0.7
08	27	Male	Ametropia (RE)	0.1	−0.25	0.3	+1.00/+0.50 × 80	0.1	0.3	0.2	0.3
09	38	Female	Anisometropia (LE)	0.6	+2.25/+0.75 × 120	0.2	−0.75	0.6	0.2	0.5	0.1
10	30	Female	Anisometropia and strabismus (RE)	0.2	+0.50/−0.75 × 70	0.4	+5.75/−2.75 × 125	0.2	0.5	0.3	0.5
11	39	Female	Anisometropia and strabismus (LE)	0.5	+0.25/+1.25 × 105	0.1	−2.50	0.4	−0.1	0.4	0
12	27	Male	Anisometropia (LE)	0.9	+5.50/+2.25 × 125	0.1	−0.75/+0.50 × 180	0.8	0.1	0.8	0.1
13	25	Male	Anisometropia (RE)	−0.1	−6.50	0.9	+1.00/0.75 × 175	−0.1	0.8	−0.1	0.8
14	23	Female	Anisometropia (LE)	0.7	+1.50/−0.50 × 65	0.1	+0.00	0.6	0.1	0.5	−0.1
15	24	Female	Anisometropia and strabismus (LE)	0.6	+2.00/1.25 × 85	−0.1	−3.50	0.6	0	0.6	0.1

*Note*: The strabismus angles of the patients in the present study were no more than 5°.

Abbreviations: LE, left eye; logMAR, logarithm of minimum angle of resolution; RE, right eye.

### 2.2 Hardware

For the adaptation task, we adopted the altered‐reality system that was used in Bao et al. (2018). The system comprised a camera (DFK‐22AUC03, USB 2.0, 640 × 480‐pixel RGB24 video recorded at 60 Hz, The Imaging Source Asia Co., Ltd., Taipei City, Taiwan) connected to a computer that fed into a head‐mounted display (HMD). The computer was a Dell Optiplex 9010MT computer with an Nvidia (Santa Clara, CA) GeForce GTX670 graphics processing unit. The HMD was Sony HMZ‐T2 (organi‐clight‐emitting‐diode display, 49.4° in horizontal, 27.8° in vertical, resolution = 1280 × 720 pixels).

### 2.3 Image acquisition and image processing during complementary‐patchwork adaptation

MATLAB (The MathWorks, Natick, MA) and Psychophysics toolbox (Kleiner et al., 2007) were used to acquire, process, and display the stimuli. The camera fixed on the altered‐reality helmet captured images of the scene in front of the participants in real time. Then, the camera image was clipped to a resolution of 640 × 360 pixels and expanded to a resolution of 1280 × 720 pixels. Subsequently, the image was divided into a 9 × 16 grid of square cells (subtending 3.1° each, see Figure 1a). Two complementary images were created from the gridded image for the two eyes. For image presented in one eye, the image in half of the cells of the gridded image was rendered uniformly in the mean color of all the pixels within that cell; the content of the remaining cells was unaltered. For image presented in the other eye, the selection of uniform cells was opposite, such that uniform cells presented to one eye corresponded to image cells presented to the other. The location of the uniform cells was randomized and updated every 10–50 s (30 s on average).

### 2.4 Procedure

All participants repeatedly adapted to complementary‐patchwork video images for 6 daily sessions. Each session lasted for 3 h. During adaptation, participants viewed the environment in front of them through the altered‐reality system. Normally, they watched videos in our laboratory room. For the amblyopia patients, visual acuity (measured with ETDRS charts) and functional imaging data were acquired before (pre‐test), 1 day after (post‐test) and about 1 month after (post 1 month, 32.15 ± 3.71 days) the 6‐day training. Due to extreme head motion, one patient repeated the fMRI post‐test on the 4th day after training. The 1‐month post‐test of one patient was canceled due to the outbreak of COVID‐19 in 2020. The healthy controls just finished the pre‐ and post‐tests due to the same reason.

### 2.5 MRI data acquisition

Participants were scanned with a Siemens 3 T Magnetom Trio scanner, using a 20‐channel phased array head coil. High resolution T1 weighted anatomical images were acquired at the beginning of each session (176 interleaved sagittal slices, repetition time [TR] = 2600 ms, echo time [TE] = 3.02 ms, flip angle = 8°, field of view [FOV] = 256 mm, voxel resolution = 1.0 mm × 1.0 mm × 1.0 mm). Resting state data were obtained after the anatomical scan with T2* weighted Echo‐planar imaging (EPI) (TR = 2000 ms, TE = 30 ms, flip angle = 90°, 32 axial slices, FOV = 200 mm, voxel resolution = 3.1 mm × 3.1 mm × 3.5 mm). The rs‐fMRI run consisted of 300 whole‐brain volumes. Participants fixed on a central white cross on the gray background during scanning.

### 2.6 Data analysis

#### 2.6.1 Preprocessing of rs‐fMRI data

The rs‐fMRI data were analyzed using AFNI software package (https://afni.nimh.nih.gov). The first two volumes were discarded for magnetization equilibrium. Then the individual's data were analyzed with the following steps of (1) outlier detection, (2) despiking, (3) slice timing correction, (4) motion correction, (5) anatomy‐to‐function image co‐registration, (6) removal of signal variations associated with head motion, motion derivatives, cerebral spinal fluid, and local white matter, (7) band‐pass filtering (0.009 < *f* < 0.08 Hz).

#### 2.6.2 Definition of ROI in visual cortex

Visual areas, including V1, V2 and V3, were identified for each participant based on anatomical templates provided by Benson (https://hub.docker.com/r/nben/occipital_atlas/) (Benson et al., 2018) on the surface of each quadrant. The ROIs were defined with a 5° gap in polar angle between areas to minimize the interactions between adjacent visual areas.

#### 2.6.3 FC within early visual areas

**FIGURE 2:**
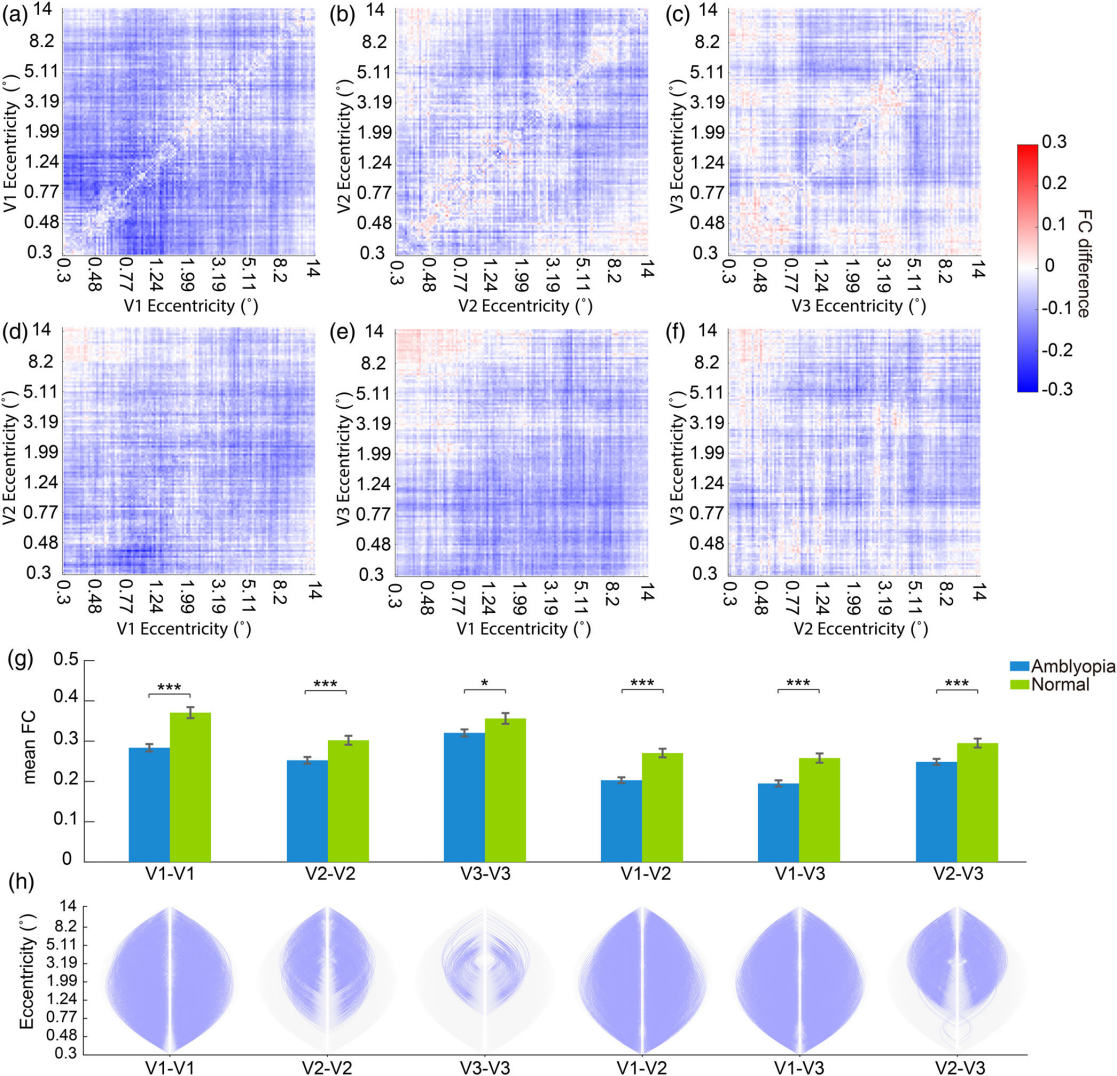
Difference of the fine‐scale FC between amblyopes and normal controls. (a–f) showed the matrices of FC difference for within or cross visual area in the pre‐test. (g) The mean FCs of the two participant groups. Significant difference between groups is denoted by asterisk (*p_FDR_ < .05, ***p_FDR_ < .001). (h) Diagram of the decreased voxel‐wise FC in amblyopes revealed by TFCE and permutation test. Grey curves represent all possible voxel‐wise connections. The significantly decreased connections are highlighted by purple curves. Note that some of the plots of connection curves appear like solid purple or gray leaf‐shapes because the connection curves are so dense.

After the pre‐processing pipeline, 4D rs‐fMRI was projected to individual's surface space. For each quadrant, the time course of each voxel in each visual ROI was extracted and sorted according to their eccentricities. Then the fine‐scale FC was analyzed within early visual areas with MATLAB and Python. Unlike the previous studies that accessed the FC between sub‐regions of visual cortex or visual cortex and other brain areas in amblyopic and normal‐sighted participants (Ding, Liu, et al., 2013; Mendola et al., 2018; Wang et al., 2014), we performed the analysis on voxel‐wise FC in early visual areas (Butt et al., 2013), which avoided any arbitrary selection and division of ROIs. We calculated the voxel‐wise FC as the Pearson correlation between every two voxels within one visual area or across two visual areas in each quadrant, yielding 6 (V1–V1, V2–V2, V3–V3, V1–V2, V1–V3 and V2–V3) × 4 (quadrants) FC matrices for each session. Because there were limited number of voxels representing the visual field at large eccentricity or the central fovea, we restricted our analysis to voxels with 0.3°–14° eccentricity. Besides, since the number of voxels of each participant and each session were not always the same, the FC matrices were aligned to a common coordinate via interpolation along eccentricity. The interpolation steps were determined according to the cortical magnification factor (Engel et al., 1997). Statistical comparison between participant groups and sessions were then performed with these matrices (Figure 2). Specifically, repeated measures analysis of variance (ANOVA) and *t*‐test were adopted to compare the mean FC. The results were corrected for multiple comparison by false discovery rate (FDR) (Benjamini & Hochberg, 1995). In addition, threshold‐free cluster enhancement (TFCE) (Smith & Nichols, 2009) and permutation test were used to identify any voxel‐wise FC that show significant difference between groups and sessions. The analysis was performed with MNE‐Python (Alexandre et al., 2013). The permutation was run 1000 times and the statistical threshold was *p* < .05.

#### 2.6.4 Exploratory analysis of FC between visual areas and other brain regions

To search in the whole brain for any change of FC with visual areas, we calculated the FC between the timeseries of each seed visual area (V1–V3) and that of each voxel in the whole brain to obtain the seed‐based FC map. The preprocessed rs‐fMRI data were smoothed (FWHM = 8 mm) and then aligned to the MNI template. Note that we did not include this step when analyzing the fine‐scale FC within early visual areas since such a smoothing process compromises spatial resolution (Mendola et al., 2018). We next performed the analysis at individual level with a multiple regression model. The mean time courses of the visual areas were extracted first. Then, the timeseries data of each visual ROI were regressed against those of each voxel of the whole brain with the variations associated with the other ROIs controlled. The fisher transformed correlation coefficients were then compared between sessions for each seed visual area respectively by paired *t*‐test. Any clusters with significant session difference were determined by Monte‐Carlo simulations with single voxel *p* < .001, cluster threshold *α* = 0.05.

#### 2.6.5 Change of visual acuity and its association with the change of FC

Repeated measures ANOVA and paired *t*‐test were used to check if there were any change of visual acuity after training. Since the impaired visual acuity and training effects were mainly observed in the results of amblyopia group (see Section 3), we then examined if there was any correlation between visual acuity and FC before training, or between the training induced improvement of visual acuity and the change of FC of amblyopes using linear regression.

## 3 RESULTS

### 3.1 Improved visual acuity of amblyopes after training

We first compared the visual acuity of amblyopia patients before and after training (N = 15, Figure 1). A 2 (Eye: amblyopic eye (AE) vs. fellow eye (FE)) × 2 (Session: pre‐test and post‐test) repeated measures ANOVA showed the significant main effect of Eye (*F*(1,14) = 91.00, *p* < .001) and Session (*F*(1,14) = 6.00, *p =* .028) along with a marginally significant interaction between Eye and Session (*F*(1,14) = 3.80, *p =* .072). Further analysis indicated that the visual acuity of AE was poorer than FE. The visual acuity improved significantly after training, especially for the AE (AE: pre‐test: 0.71 ± 0.21 logMar, post‐test: 0.64 ± 0.17 logMar, *t*(14) = 3.16, *p =* .007; FE: pre‐test: 0.07 ± 0.15 logMar, post‐test: 0.06 ± 0.15 logMar, *t*(14) = 0.62, *p =* .546). Then we investigated whether the training effect was long‐lasting based on the results of patients who had completed the follow‐up test (*N* = 14). Surprisingly, we found the improvement of visual acuity was further enhanced in the following month after training (pre‐test: 0.71 ± 0.22 logMar, post‐test: 0.64 ± 0.18 logMar, post 1 month: 0.61 ± 0.18 logMar, post‐test vs. post 1 month: *t*(13) = 2.28, *p =* .040).

As some of the patients were strabismic amblyopes and some were anisometropic, we then performed an exploratory analysis to compare the training effect between the two samples. Training improved the visual acuity for the AE in 2 out of 5 strabismic patients and 7 out of 10 anisometropic patients. However, perhaps due to low sample size of each group, independent sample *t*‐test on the acuity change of AE from the pre‐ to post‐test showed no significant group difference (*t*(14) = 0.89, *p* = .391). Since the strabismic amblyopes only had mild strabismus (no more than 5° of strabismus angle) and all reported good binocular fusion during adaptation, the two amblyopia groups are thought to have indistinguishable training effects. Therefore, we did not separate the two samples in the following analysis.

In comparison with the amblyopes, the visual acuity of normal‐sighted participants remained constant before and after training (pre‐ vs. post‐test: left eye: *t*(13) = 0.27, *p =* .793; right eye: *t*(13) = 0.81, *p =* .435).

### 3.2 Reduced fine‐scale FC in early visual areas of amblyopes

Compared to normal‐sighted participants, in the pre‐test, the voxel‐wise FCs of amblyopia patients were reduced both within and across visual areas (Figure 2, V1–V1: *t*(113) = −5.37, *p* < .001, *p*_FDR_ < .001; V2–V2: *t*(113) = −3.62, *p* < .001, *p*_FDR_ < .001; V3–V3: *t*(113) = −2.28, *p* = .024, *p*_FDR_ = .024; V1–V2: *t*(113) = −5.30, *p* < .001, *p*_FDR_ < .001; V1–V3: *t*(113) = −4.59, *p* < .001, *p*_FDR_ < .001; V2–V3: *t*(113) = −3.53, *p* < .001, *p*_FDR_ < .001).

We next performed the detailed analysis on the voxel‐by‐voxel FCs using TFCE and permutation test. Significantly decreased cross‐eccentricity FCs in amblyopes as compared to normal controls are denoted as purple curves in Figure 2h. No enhanced FCs were found. The results indicated that the FCs within V1 and between V1 and V2 or V3 were generally decreased in amblyopia. In addition, the decrease of FCs within V2/V3 or between V2 and V3 were more pronounced between voxels representing the central visual field and those representing the periphery.

### 3.3 Selective enhancement of fine‐scale FC within early visual areas of amblyopes after training

**FIGURE 3:**
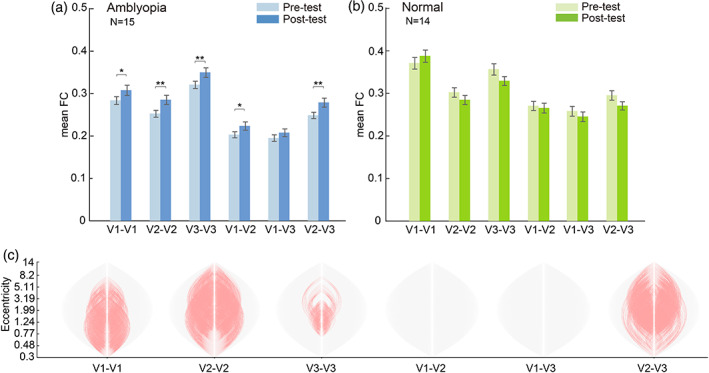
Voxel‐wise FC within and between visual areas in different sessions. (a) Mean voxel‐wise FC of the amblyopia group. Significant difference between sessions is denoted by asterisk (*p_FDR_ < .05, **p_FDR_ < .01). (b) Results of the control group. (c) Diagrams of the change of voxel‐wise connections within and between visual areas of amblyopes. The significantly enhanced connections are denoted by red curves.

After the six daily sessions of complementary patchwork adaptation, we found that the fine‐scale FC increased significantly within the early visual cortex of amblyopia patients (Figure 3a, *N* = 15, V1–V1: *t*(59) = 2.49, *p* = .016, *p*_FDR_ = .023; V2–V2: *t*(59) = 3.76, *p* < .001, *p*_FDR_ = .002; V3–V3: *t*(59) = 2.97, *p* = .004, *p*_FDR_ = .009; V1–V2: *t*(59) = 2.14, *p* = .036, *p*_FDR_ = .044; V1–V3: *t*(59) = 1.08, *p* = .284, *p*_FDR_ = .284; V2–V3: *t*(59) = 3.309, *p* = .002, *p*_FDR_ = .005). Detailed analysis on the voxel‐wise FC showed increased central‐periphery connections within V1, V2, V3 and between V2 and V3 (Figure 3c).

**FIGURE 4:**
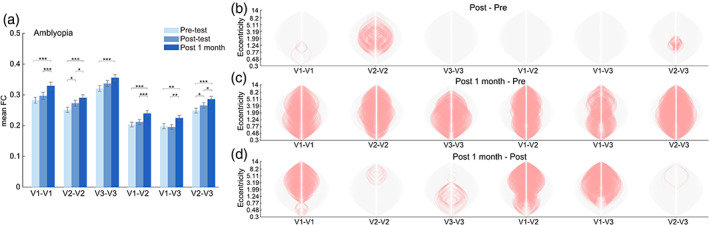
FC of amblyopes in three test sessions (*N* = 14). (a) Mean voxel‐wise FC of each session. (b–d) The change of voxel‐wise connections within and between visual areas. Each plot represents the contrast between post‐test and pre‐test, post 1 month test and pre‐test, post 1 month test and post‐test, respectively.

Similar to the results of visual acuity, further enhancement of FC was observed in the follow‐up test (*N* = 14, Figure 4a, Table 2). By comparing the difference of voxel‐wise FC between sessions, we found the FCs were enhanced generally across most of eccentricities a month after training (Figure 4b–d).

**TABLE 2:** Statistics of *t*‐test comparison of the session difference of the mean FC from amblyopes.

	Post vs. pre		Post 1 month vs. pre		Post 1 month vs. post	
	*t* (df = 55)	*p*_FDR_	*t* (df = 55)	*p*_FDR_	*t* (df = 55)	*p*_FDR_
V1–V1	1.61	.170	5.81	<.001	3.96	<.001
V2–V2	3.10	.018	5.55	<.001	2.20	.038
V3–V3	2.11	.078	3.83	<.001	1.81	.075
V1–V2	1.04	.361	4.63	<.001	3.90	<.001
V1–V3	−0.23	.822	2.97	.004	3.38	.003
V2–V3	2.60	.036	5.20	<.001	2.33	.035

In contrast, results from the control group indicated no significant change of FC after training (Figure 3b, V1–V1: *t*(55) = 1.34, *p* = .186, *p*_FDR_ = .279; V2–V2: *t*(55) = −1.37, *p* = .176, *p*_FDR_ = .279; V3–V3: *t*(55) = −2.11, *p* = .039, *p*_FDR_ = .162; V1–V2: *t*(55) = −0.43, *p* = .670, *p*_FDR_ = .670; V1–V3: *t*(55) = −1.11, *p* = .272, *p*_FDR_ = .327; V2–V3: *t*(55) = −1.97, *p* = .054, *p*_FDR_ = .162).

### 3.4 Association between visual acuity and FC of amblyopes

**FIGURE 5:**
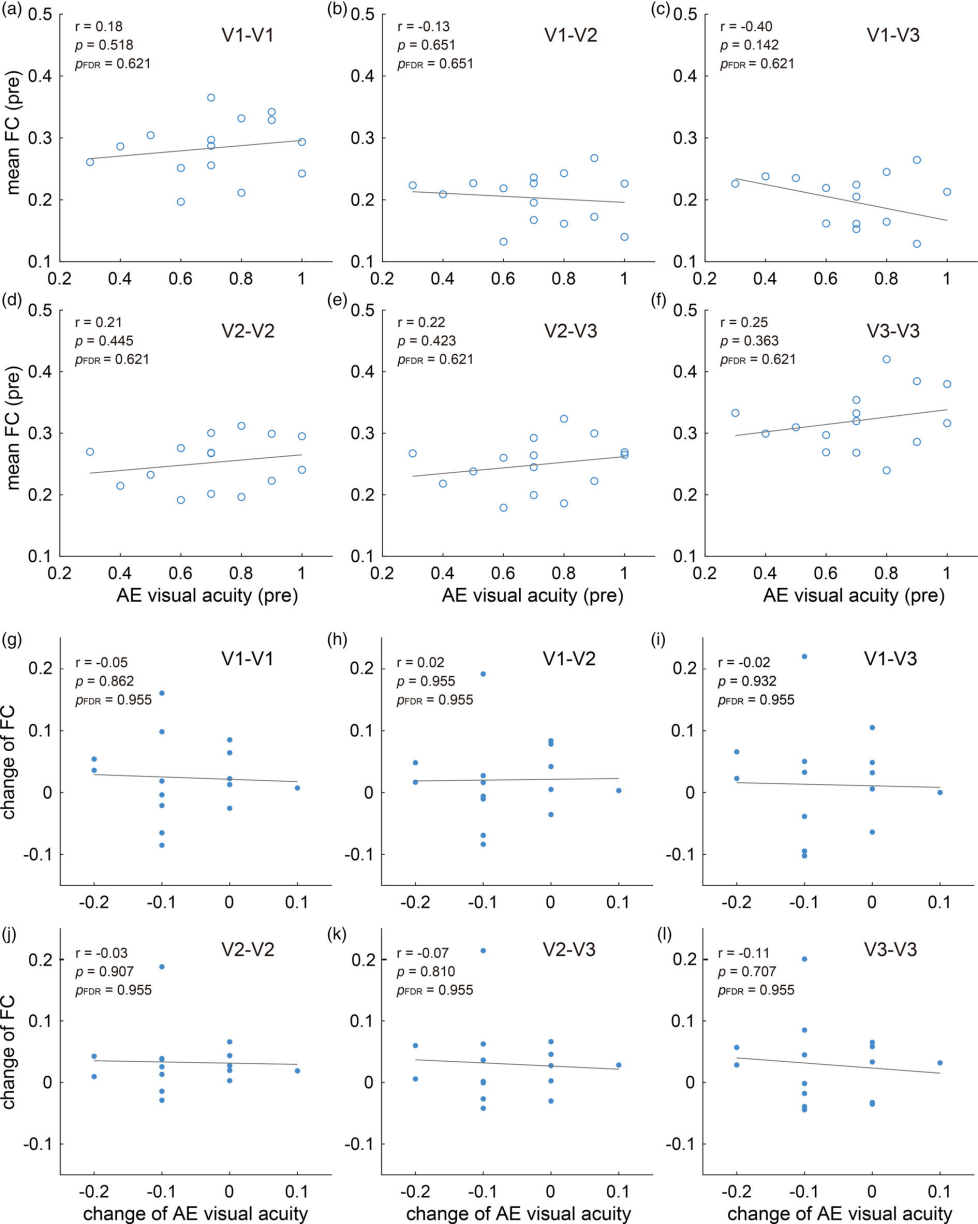
Correlation between visual acuity and FC. (a–f) show the correlation between visual acuity and mean FC within or across visual areas before training. (g–l) show the correlation between training induced changes of visual acuity and the altered FC.

Linear regression was performed to explore the association between the impaired AE acuity and FC or the association between the training induced change of AE acuity and that of FC. However, we did not find significant correlations between visual acuity and the FC (Figure 5).

### 3.5 FC between visual ROIs and other brain regions

We tried seeking any inter‐session difference on the FC between each visual ROI and other brain areas in the whole brain, but did not find any significant clusters that survived the correction of multiple comparison in either participant group. Therefore, our findings suggested that the altered FC induced by training was limited within the local visual areas and was specific to the amblyopes.

## 4 DISCUSSION

Using a complementary‐patchwork adaptation paradigm, we asked one group of patients with amblyopia and one group of healthy control participants to complete a 6‐day training. This adaptation training led to a long‐term boost of visual acuity in patients with amblyopia, together with the enhanced voxel‐wise FCs in early visual areas. Both the visual acuity and the fine‐scale FCs continued to be enhanced in the following 1 month after the training ended. By contrast, such effects were not observed in the control group though they underwent the same training.

Improved visual acuity marked the amelioration of amblyopes' visual functions, though the abnormal visual functions in amblyopes are far more than poor visual acuity. Before the training, we found that the fine‐scale voxel‐wise FCs in early visual areas of amblyopes were much weaker than those of normal controls, extending our understanding about the abnormal functional connections in amblyopia. More importantly, our work for the first time revealed that the brain FC in amblyopia was ameliorated after an effective training, and further enhanced in the subsequent month where the patients lived in everyday life. One possible mechanism for the increasing cross‐eccentricity FC is that the adaptation paradigm improved the integration of dichoptic information on adjacent visual field and thus increased the cross‐eccentricity interaction. To form an intact image while viewing the complementary‐patchwork images, the visual system should integrate the square cells that contain normal images in the two eyes. The improved interocular integration is to some extent supported by the subjective reports of many patients that they could have more intact view and better fusion after a few days of adaptation. In addition, our training also enhanced the connectivity across V1–V3, which is previously found to be attenuated in amblyopia when compared with normal‐sighted participants (Mendola et al., 2018), and is thought to reflect the reduced feedforward transmission in the hierarchical visual pathway. Thus, the findings in our study imply that the adaptation training could also promote the hierarchical connections.

Alternatively, the increased voxel‐wise FC may serve as a compensatory mechanism against the amblyopia‐associated visual deficits, especially since such an effect was only observed in the results of the patient group. The potential compensatory mechanism has been reported in studies that found increased regional homogeneity in somatosensory cortex, motor areas and auditory areas (Lin et al., 2012) or increased long range brain FC between the primary visual area and postcentral gyrus (Ding, Liu, et al., 2013) in amblyopia. Our findings suggest that similar compensatory plasticity may also exist in local visual network. Such compensatory functional adjustment can be evoked by our training method, and continue to develop in the subsequent month of living in the normal visual environment.

Interestingly, the visual acuity of amblyopes also continued to improve in the following month after the end of adaptation training, replicating our previous finding (Bao et al., 2018). Although the underlying mechanism remains unclear, we embrace the notion (Bao et al., 2018) that several days of adaptation training may reduce the suppression of signals from the amblyopic eye by those from the fellow eye, and that such disinhibition is long‐lasting, allowing the amblyopic eye to continue strengthening through natural learning in subsequent everyday life. This account has received some supports from both the finding of training‐induced persistent (≥8 weeks) attenuation of the Ebbinghaus illusion (Bao et al., 2018), which presumably indicates weakened suppression in the visual cortex (Lunghi et al., 2015; Tibber et al., 2013; Yoon et al., 2010), and the finding of long‐lasting (≥1 month) reduction of intermodulation steady‐state visually‐evoked‐potential response following training (Du et al., 2023), which is thought to reflect decreased interocular suppression (Katyal et al., 2016, 2018; Suter et al., 1996). Remarkably, the changing pattern of the fine‐scale FC within early visual areas was in good concordance with that of visual acuity. As shown in Figure 4, the early visual areas showed even greater enhancement of fine‐scale FCs in the post‐1‐month test compared to the post‐test. The similar temporal dynamics of visual acuity and fine‐scale FCs may thus hint some common underlying mechanisms. For instance, training might first produce disinhibition, which then evokes the mechanisms responsible for the improvement of visual acuity and enhancement of fine‐scale FC.

The present results did not show any changes of long‐range FCs between the visual cortex and other brain areas after training. As it is suggested in Sireteanu (1982) and Sireteanu and Fronius (1981), amblyopia is likely to be the consequence of long‐term suppression, the abnormal cross‐network FC in amblyopes may also result from years of the impaired information transmission and chronically abnormal visual input. Therefore, we presume that improving this type of FC likely requires longer‐term training.

We did not find any correlation between the FC and visual acuity before training or the altered FC and the improved visual acuity after training. In fact, literature never reaches an agreement on whether there should always exist a correlation between impaired visual acuity and altered FC in amblyopes. Mendola et al. (2018) analyzed the FC between nine subregions of V1–V3 divided according to eccentricity (e1–e3 from fovea to periphery) and only observed a correlation between the FC of peripheral subregions of V1 (V1e3–V1e2) and visual acuity. Besides Mendola et al.'s work, the studies from two other groups also investigated the FC in amblyopes between the primary visual cortex and other brain regions or between different visual networks (Ding, Liu, et al., 2013; Lu et al., 2019). However, no significant correlation was found between the altered FC and disease severity (visual acuity). Thus, it seems that impairment of visual acuity alone did not always correspond to cortical defects of FC. As we have known that visual acuity is not the only visual function improved by the present adaptation training (Bao et al., 2018; Du et al., 2023), it is possible that the altered FC is relatively a local functional change that could in some way contribute to, though do not largely overlap with, the mechanisms related to the increase of visual acuity. Since fMRI usually represents mass brain activation, the observation from rs‐fMRI may not coincidentally and directly reflect the measured behavioral results. In addition, the limited sample size in our study may also impede the detection of correlations.

In conclusion, our study again confirmed that the complementary‐patchwork adaptation is an effective method to treat amblyopia even in adults. More important, we found that such adaptation training enhanced the voxel‐wise FC within the early visual areas, providing new insight into the neural plasticity of adult visual cortex. Decreased brain FC has been commonly observed in patients with amblyopia, which is often considered to reflect their impaired visual functions and deficits of brain functional coordination. The present work for the first time reveals that effective treatment can improve both the adult amblyopes' vision and brain functional connections in the visual cortex, setting a vivid example of how spontaneous brain activity at rest can be long‐term reshaped by repetitively viewing augmented reality (Bao & Engel, 2019). Future work is expected to explore the specific visual functions that benefit from the training‐induced enhancement of FC.

## CONFLICT OF INTEREST STATEMENT

Min Bao holds a patent on an invention for using an altered‐reality system to rebalance the ocular dominance. All authors declare no other potential conflicts of interest with respect to the authorship or the publication of this article.

## Data Availability

The data that support the findings of this study are available from the corresponding author upon reasonable request.
